# Attenuated post-movement beta rebound reflects psychomotor alterations in major depressive disorder during a simple visuomotor task: a MEG study

**DOI:** 10.1186/s12888-023-04844-3

**Published:** 2023-06-03

**Authors:** Yi Xia, Lingling Hua, Zhongpeng Dai, Yinglin Han, Yishan Du, Shuai Zhao, Hongliang Zhou, Xiaoqin Wang, Rui Yan, Xumiao Wang, HaoWen Zou, Hao Sun, YingHong Huang, ZhiJian Yao, Qing Lu

**Affiliations:** 1 Department of Psychiatry, the Affiliated Brain Hospital of Nanjing Medical University, Nanjing, 210029 China; 2 School of Biological Sciences & Medical Engineering, Southeast University, Nanjing, 210096 China; 3 Child Development and Learning Science, Key Laboratory of Ministry of Education, Southeast University, Nanjing, 210096 China; 4 Nanjing Brain Hospital, Medical School of Nanjing University, Nanjing, 210093 China

**Keywords:** Magnetoencephalography, Major depressive disorder, Primary motor cortex, Psychomotor alterations, Post-movement beta rebound

## Abstract

**Background:**

Psychomotor alterations are a common symptom in patients with major depressive disorder (MDD). The primary motor cortex (M1) plays a vital role in the mechanism of psychomotor alterations. Post-movement beta rebound (PMBR) in the sensorimotor cortex is abnormal in patients with motor abnormalities. However, the changes in M1 beta rebound in patients with MDD remain unclear. This study aimed to primarily explore the relationship between psychomotor alterations and PMBR in MDD.

**Methods:**

One hundred thirty-two subjects were enrolled in the study, comprising 65 healthy controls (HCs) and 67 MDD patients. All participants performed a simple right-hand visuomotor task during MEG scanning. PMBR was measured in the left M1 at the source reconstruction level with the time–frequency analysis method. Retardation factor scores and neurocognitive test performance, including the Digit Symbol Substitution Test (DSST), the Making Test Part A (TMT-A), and the Verbal Fluency Test (VFT), were used to measure psychomotor functions. Pearson correlation analyses were used to assess relationships between PMBR and psychomotor alterations in MDD.

**Results:**

The MDD group showed worse neurocognitive performance than the HC group in all three neurocognitive tests. The PMBR was diminished in patients with MDD compared to HCs. In a group of MDD patients, the reduced PMBR was negatively correlated with retardation factor scores. Further, there was a positive correlation between the PMBR and DSST scores. PMBR is negatively associated with the TMT-A scores.

**Conclusion:**

Our findings suggested that the attenuated PMBR in M1 could illustrate the psychomotor disturbance in MDD, possibly contributing to clinical psychomotor symptoms and deficits of cognitive functions.

## Background

Major depressive disorder (MDD) is not only characterised by abnormalities in mood and affect but also substantial disturbance in cognition and psychomotor function. Psychomotor alterations have been recognised as one of the crucial features of MDD [1]. The term psychomotor is composed of two parts: "psycho" and "motor," with the former referring to cognitive processes and the latter to motor processes [2]. Psychomotor functions are the foundation of the plan and execution of various behaviour, including speech, facial expression, and cognitive function [3]. Some findings showed that one aspect of psychomotor alterations, namely psychomotor retardation (PMR), is related to the severity of depression [4] and poor response to antidepressants [5], ultimately contributing to impaired social function and quality of life [6]. Understanding the neuronal basis of PMR could therefore ultimately contribute to development of treatments permitting improved quality of life.

Despite this importance, research on psychomotor issues in MDD is quite scarce. Previous work has primarily probed the pathophysiology of psychomotor alterations in mood disorders [7]. It has been suggested that the mania state in mood disorders is associated with psychomotor excitation, while the depressive state is mainly related to psychomotor inhibition [8]. A small handful of studies have found that the psychomotor function is modulated by the sensorimotor network [2, 9]. It was suggested that the psychomotor alterations in patients with MDD are correlated with neural circuitry involving the prefrontal cortex, motor-related cortex and basal ganglia [1]. In particular, PMR is due to the alteration of cerebellar-thalamo-cortical motor circuitry [10]. Existing functional imaging studies have demonstrated that psychomotor alterations are related to decreased blood flow in the dorsolateral prefrontal cortex, left prefrontal cortex, angular gyrus, and anterior cingulate [11–13]. Furthermore, structural imaging studies revealed that psychomotor speed is associated with alterations of white matter in motor-related pathways [14–16]. A study discovered that aberrant cerebral blood flow (CBF) in the centre of the motor-regulating circuit might account for the PMR in MDD using a perfusion MRI approach [17]. Therefore, structural and functional alterations in the motor-related cortex might result in the PMR in MDD. Although the engagement of sensorimotor cortices in psychomotor function is preliminarily documented, the specific brain activity pattern remains ambiguous.

Magnetoencephalography (MEG) allows non-invasive inference of current flow in neuronal cell assemblies through measurement of extracranial magnetic fields. Due to its high temporal resolution, MEG is ideally suited to capturing neural activity during psychomotor processes. A well-characterised neurophysiological feature associated with cognition and motor control is beta-band oscillations [18]. They play a vital role in long-range communication on a cortical network level and in particular, mediate feedback influences through which higher brain centres influence centres lower in the processing hierarchy [19]. Based on previous research, MDD is characterised by dominant beta oscillations [20, 21]. In our initial studies, spontaneous central beta power might be a potential biomarker of MDD [22, 23]. Moreover, we found that resting-state beta-band oscillations are related to response speed [24] and process speed [25]. These investigations have advanced our understanding of the impact of MDD on sensorimotor cortical oscillations. However, due to a lack of task-based evidence, motor-related beta oscillation pattern and the relevance of brain abnormalities in motor systems to psychomotor disturbances in MDD remain largely unknown.

**Fig. 1:**
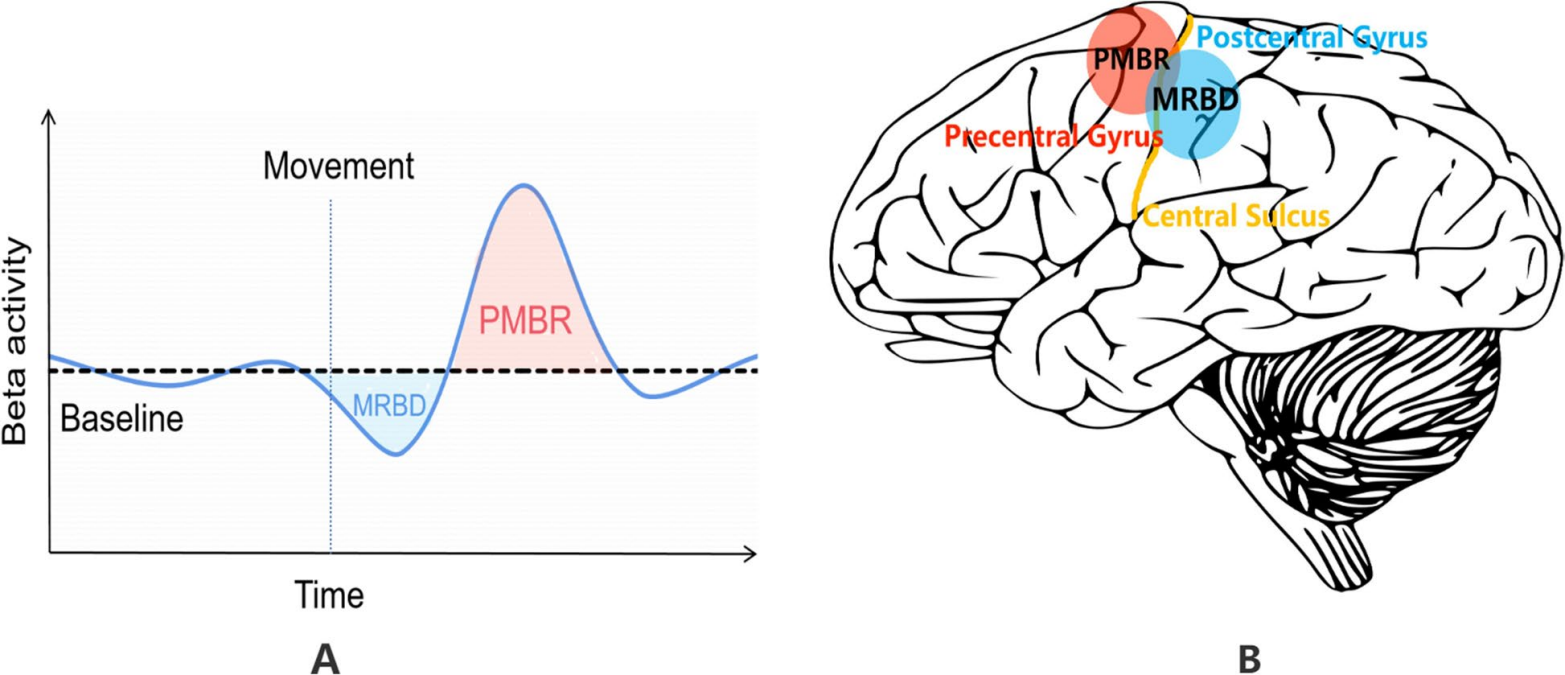
Illustration of MRBD and PMBR. **A** Schematic representation of sensorimotor beta activity. Beta activity reduces (MRBD—blue shaded region) right before and during movement execution. After the end of movement, beta activity rapidly increases (PMBR—red shaded region). Finally, beta activity slowly returns to baseline level. **B** The brain distribution of MRBD and PMBR. MRBD (blue circle) is frequently found in the postcentral gyrus, while PMBR (red circle) is frequently observed in the precentral gyrus

As depicted in Fig. 1, a power reduction in beta bands across sensorimotor regions is present before movement onset and during movement execution, i.e., movement-related beta reduction (MRBD) [19]. Following the end of the movement, post-movement beta rebound (PMBR) occurs, defined as an increase in beta amplitude relative to baseline [26]. This response typically occurs 500 ms after movement termination and lasts for several seconds (Fig. 1A). Previous studies have suggested that the PMBR originates in the sensorimotor network [18], particularly M1 [27]. There is evidence that MRBD and PMBR may originate from different brain regions. Specifically, MRBD is usually located in the postcentral gyrus, while PMBR locates in the precentral gyrus (Fig. 1B) [27, 28]. Moreover, the two stage-dependent alterations may play different roles in sensorimotor processes. As PMBR is well-accepted to be involved in motor inhibition [29] or sensory feedback [30] in motor cortices, it could regulate cortical sensory processing and motor output [31], which further supports the notion that sensorimotor beta modulation may be a key marker to investigate psychomotor process [32]. Moreover, PMBR may contribute to visuomotor adaption by maintaining or updating the forward model that guides movement [33]. Altogether, previous neurophysiological studies have highlighted that the strength of motor-related beta oscillations is directly related to cognitive-motor performance.

Research in healthy individuals demonstrated that the decrease of PMBR leads to a slower movement state [34], consistent with reduced movement-related beta activity in Parkinson's disease (PD) [35]. Many physiological or pathological factors influence beta oscillations, such as healthy ageing [36–45]. Previous studies found that poor motor performance in healthy older adults is related to changes in beta oscillations. Importantly, neuropsychiatric studies also revealed that decreased PMBR in PD [46–48], SCH [49–51], schizotypal personality disorder (SPD) [52], autism spectrum disorder (ASD) [53–55], reflecting impaired sensorimotor integration in psychiatric disease. In general, these findings indicated that the aberrant PMBR impacts motor-related activity and advanced sensorimotor integration. Despite some studies reporting this movement-related neurophysiological modulation in clinical and non-clinical populations, the alteration of PMBR and its association with psychomotor disturbance in MDD remains underexplored.

In this study, we specifically addressed PMR in MDD using MEG technique combined with experimental motor tasks, clinical ratings of psychomotor disturbance, and neuropsychological tests involving psychomotor speed. We aimed to investigate the abnormal beta oscillations pattern in MDD and its potential association with psychomotor alterations. Based on available evidence, we hypothesised that patients with MDD have attenuated PMBR, and this change could reflect a psychomotor slowing in clinical symptoms and cognitive functions.

## Methods

### Study design

This study adopted a cross-sectional case–control study design. Data, including demographic data, clinical assessments, neurocognitive assessments, MEG scan data and MRI scan data, were collected from patients with MDD and healthy controls (HCs).

### Participants

The MDD patients were recruited from the Affiliated Brain Hospital of Nanjing Medical University between 2012 and 2021. All patients met the diagnostic criteria of major depressive disorder using the Diagnostic and Statistical Manual of Mental Disorders Fourth Edition, Text Revision (DSM-IV-TR). The inclusion criteria for this population were: (1) aged 18–50 years; (2) right-handed; (3) native Han Chinese; (4) education level of junior high school or above; (3) Hamilton Rating Scale for Depression 17-item (HAMD_17_) > 17; (4) Young Mania Rating Scale (YMRS) [56] score < 5; (5) no structured psychotherapy or physical therapy within six months. The exclusion criteria included comorbidity with other mental or physical illnesses, history of substance abuse, contraindications for MEG or MRI and pregnancy or lactation.

One hundred thirty-two individuals, comprising 65 healthy controls and 67 MDD patients, were enrolled in this study. Participants were asked to perform a simple visuomotor task during MEG scanning. This work was approved by the Affiliated Brain Hospital of Nanjing Medical University. It was performed in accordance with the Declaration of Helsinki, and all individuals provided written informed permission before participation.

### Clinical assessments

We collected general participant information, such as sex, age, education, ethnicity, dominant hand, disease history, marriage and family history. Depression severity was assessed using HAMD_17_, and manic symptoms were evaluated by YMRS. Based on factorial analysis found in Bertelli and the Istituto Superiore di Sanità (Italy 1977), and in the HAMD_17_ [57, 58], five structural factors included: (I) anxiety/somatization; (II) body weight; (III) cognitive disturbances; (IV) retardation; (V) sleep disturbances. Retardation factor measurement included depressed mood, work and interests, retardation and genital symptoms.

### Neurocognitive assessments

Based on previous work, psychomotor alterations could be measured by neuropsychological tests [7]. In this study, psychomotor performance was measured by three different neuropsychological taskes:1) DSST, which measures both cognitive and motor elements of psychomotor by instructing subjects to fill in as many blanks as possible within 90 s according to the number symbol correspondence table. Test performance is evaluated by the number of successful completions [59]; 2) TMT-A, a test in which individuals are directed to draw lines through consecutive numbers to test processing speed. The time taken to complete the test represents task performance [60]; and 3) VFT, a task in which participants are asked to name as many animals or vegetables as possible in 60 s examining mental processing speed [61]. These tests can accurately quantify psychomotor changes in MDD patients.

### MEG scanning

Subjects were placed in a magnetically shielded room during the entire experiment. Data were collected using a 275-channel CTF system (VSM Med Tech Inc., Port Coquitlam, Canada). The sampling rate is 1,200 Hz. The participants were lying in the MEG machine during a visuomotor task. Three coils, one nasion and two preauricular points were used to check head movements during the recording. Electrocardiography and electrooculography were also recorded. The experiment consists of two blocks, each lasting 5 min. Participants can take a small break between blocks.

### Task paradigm

**Fig. 2:**
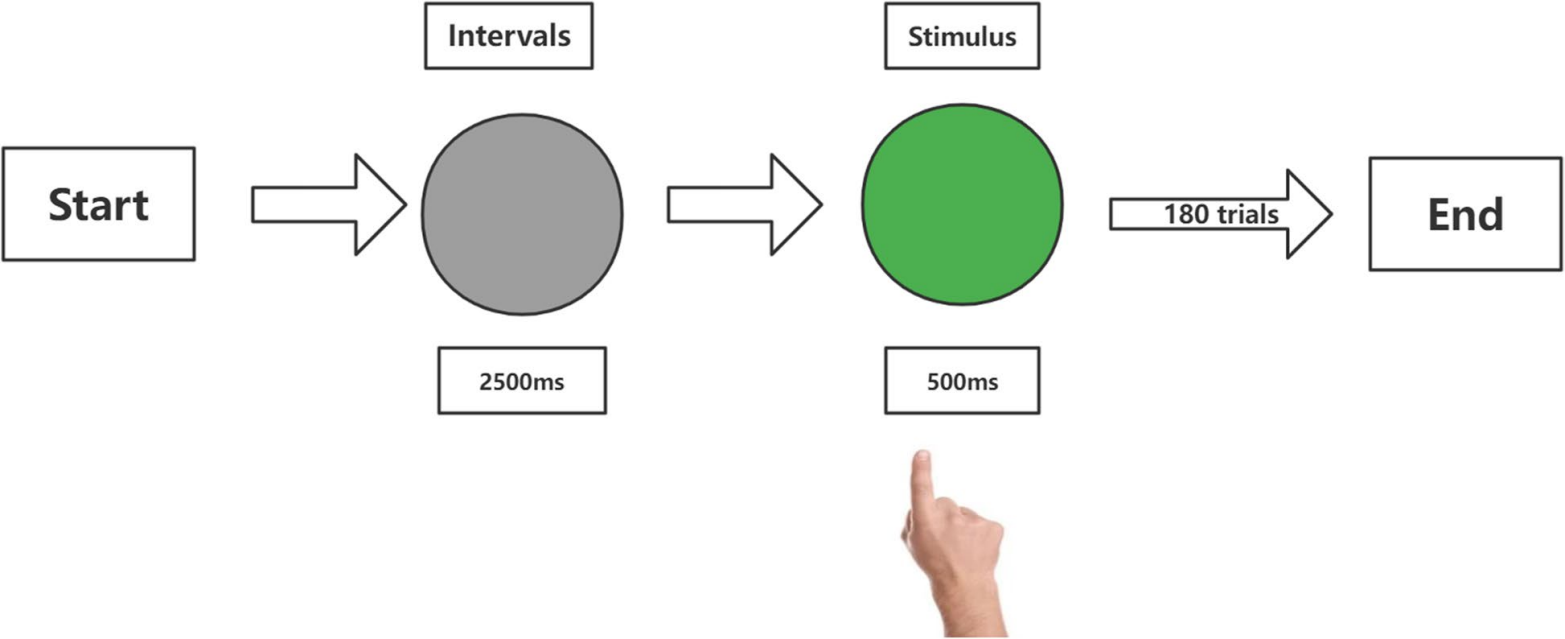
The procedure of the visuomotor experiment. A green light was presented duration of 0.5 s. Once the green light appeared, participants were asked to press the button with the right index finger. Green light was followed by an interval of 2.5 s (grey light)

Participants were asked to perform a visuomotor task during MEG scanning. Participants responded with a right index finger button press to visual stimuli. Figure 2 shows an illustration of this paradigm. During each trial, grey light was exhibited on a projection screen for 2,500 ms, then a green light lasting 500 ms. Subjects responded with a right index finger button press to visual stimuli. Individuals were asked to concentrate on the projection screen, keep their bodies still, and press the button as quickly as possible. Participants first completed a practice examination to understand the task, followed by a formal exam including 180 trials.

### MRI scanning

MRI data were recorded with a Siemens Verio 3 T MRI system. The parameters were the same as those used in our earlier articles [62]. Three markers were put in the same location as MEG to facilitate registration between MEG data and structural MRI in the following data analysis.

### Data processing

**Fig. 3:**
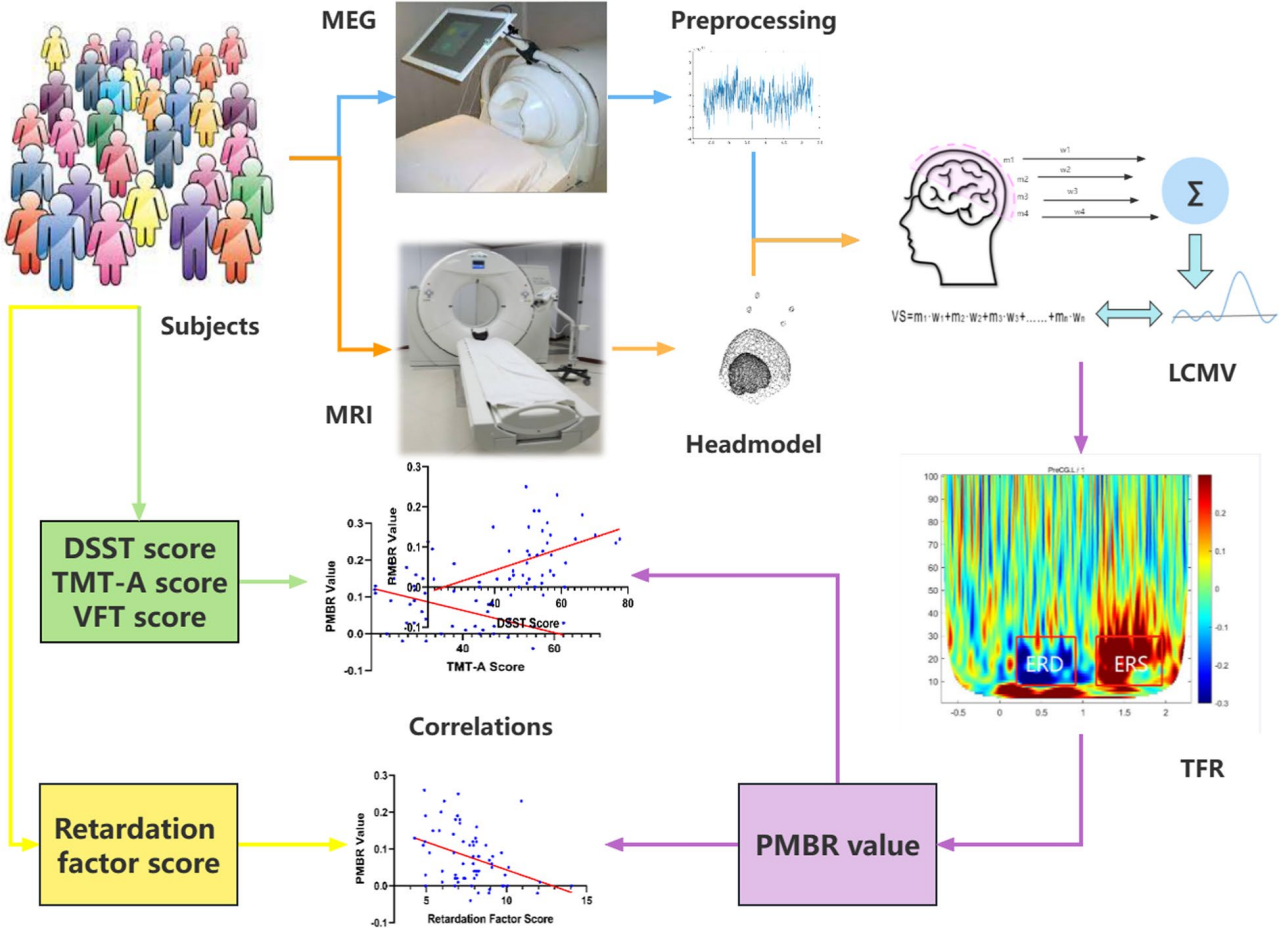
Diagram of the processing flow. For each person, we obtained three types of data, including MRI, MEG, and neurocognitive tests. DSST, TMT-A and VFT are included. For patients with MDD, retardation factor scores were also extracted. MEG data preprocessing contained segmentation, down-sampling, visual artifact rejection and independent component analysis. The headmodels of each participant were obtained by an anatomical MRI scan. Then Using the LCMV beamformer, we then extracted the time course of the left M1 defined via the AAL template atlas. We subsequently calculated the source-level TFR of this virtual sensor timeseries. By averaging the relative power of beta within a time window of interest, a single value was computed to represent the amplitude of PMBR for each participant. Finally, we assessed the relationship between PMBR and retardation factor scores, and neurocognitive task performance

The entire data processing process is illustrated in Fig. 3. The raw data were epoched into a time window (-0.7 to 2.3 s). The 0 s represent the emergence of a visual cue or green light. The data were down-sampled to 400 Hz. Then we applied Synthetic third-order gradiometer noise cancellation and removed linear trend and line interference with a 50-Hz band-stop filter. Visual artifact rejection was used to exclude trials and channels with high variation. Furthermore, with a visual examination, an independent component analysis (ICA) was used to eliminate eye artifacts, as well as cardiac and muscular components.

We analysed the MEG data in FieldTrip (version 20,210,720) [63]. The structural T1 data was imported and segmented using FieldTrip, with 250 anatomical volumes in each direction and “singleshell”.

### Beamforming and time course extraction

As PMBR is located in the contralateral M1, we want to extract the beta time series of the M1 in this right finger tap task. Using the linearly constrained minimum variance (LCMV) beamformer [64], we can compute virtual channel time series at the locations of interest. The coordinates of the M1 were defined by the Automated Anatomical Labeling (AAL) template atlas [65]. The spatial filter was computed with the covariance and the forward model for the left M1. Using the spatial filters, virtual channel time series were obtained.

### Time–frequency spectrograms

Using a Hanning taper-based time window of four cycles at each frequency, the source-level time–frequency representation of the time series of the M1 may be obtained (1–100 Hz in steps of 1 Hz). We compared the active power to the baseline using relative percentage. The baseline was defined between the -0.5–0 s window relative to the appearance of green light. The time–frequency spectrograms were averaged across participants in both groups.

### PMBR quantification

We averaged mid-beta band (17–25 Hz) amplitude from the data baseline adjusted in time windows (1 < t < 2 s) acceptable for the PMBR. Eventually, we obtained a value per participant representing the PMBR at the left M1.

### Statistical analyses

A two-sample t-test was used to check age and education, whereas chi-square tests were conducted to compare gender between MDD patients and HCs. All statistical analyses were done on SPSS 19.0 software (IBM Corp., Armonk, NY, USA). The differences in neurocognitive test performance (DSST, TMT-A and VFT) between the MDD and HC groups were compared by a two-sample t-test (two-tailed). The difference in PMBR amplitude between groups was analysed using a two-sample t-test. We then computed correlations between PMBR values and retardation factor, neurocognitive tests performance (DSST, TMT-A and VFT), controlling for age, sex and education. To avoid multiple comparison problems, further FDR correction was conducted using a special MATLAB function.

## Results

### Demographics and clinical characteristics

Sixty-seven MDD patients and sixty-five HCs were enrolled in the study. There was no significant difference in sex, age or education between the two groups. HAMD_17_ total score and retardation factor score were recorded for patients only (Table 1).

**Table 1:** Subjects’ demographic and clinical characteristics

Variables	MDD (*n* = 67)	HC (*n* = 65)	*t/χ*	*p*
Sex (M/F)	33/34	34/31	0.123^a^	0.726^a^
Age, y	30.19 ± 10.24	29.22 ± 6.48	-0.658^b^	0.512^b^
Education, y	13.90 ± 2.79	14.22 ± 2.17	0.737^b^	0.462^b^
Handedness (R/L)	67/0	65/0	-	-
HAMD_17_ total score	24.10 ± 5.58	-	-	-
Retardation	7.73 ± 1.86	-	-	-

*Abbreviations*: *MDD* Major depressive disorder, *HC* Health control, *HAMD* Hamilton Depression Rating Scale

^a^The *χ*/*p* value was obtained by two-tailed Pearson chi-square test

^b^Data were presented as the range of minimum–maximum (mean ± SD). The *t*/*p* value was obtained by two-sample two-tailed t test

### Neurocognitive tests performance

The MDD group showed worse neurocognitive test performance than controls in all three tasks (Table 2). Statistical analyses revealed significant differences in DSST (*t* = 3.05, *p* = 0.003), TMT-A (*t* = -3.38, *p* = 0.001), and VFT (*t* = 3.13, *p* = 0.003).

**Table 2:** Neurocognitive test performance and PMBR

	MDD	HC	*t*	*p*
DSST	51.91 ± 11.21	58.41 ± 5.60	3.05	0.003
TMT-A	36.40 ± 12.71	26.21 ± 8.60	-3.38	0.001
VFT	18.56 ± 4.99	22.91 ± 5.89	3.13	0.003
PMBR	0.08 ± 0.07	0.14 ± 0.12	3.70	< 0.001

Two-sample t-test, two-sided, alpha-level 0.05

*DSST* Digit Symbol Substitution Test, *TMT-A* Trail Making Test A, *VFT* Verbal Fluency Test

### Group differences of PMBR

**Fig. 4:**
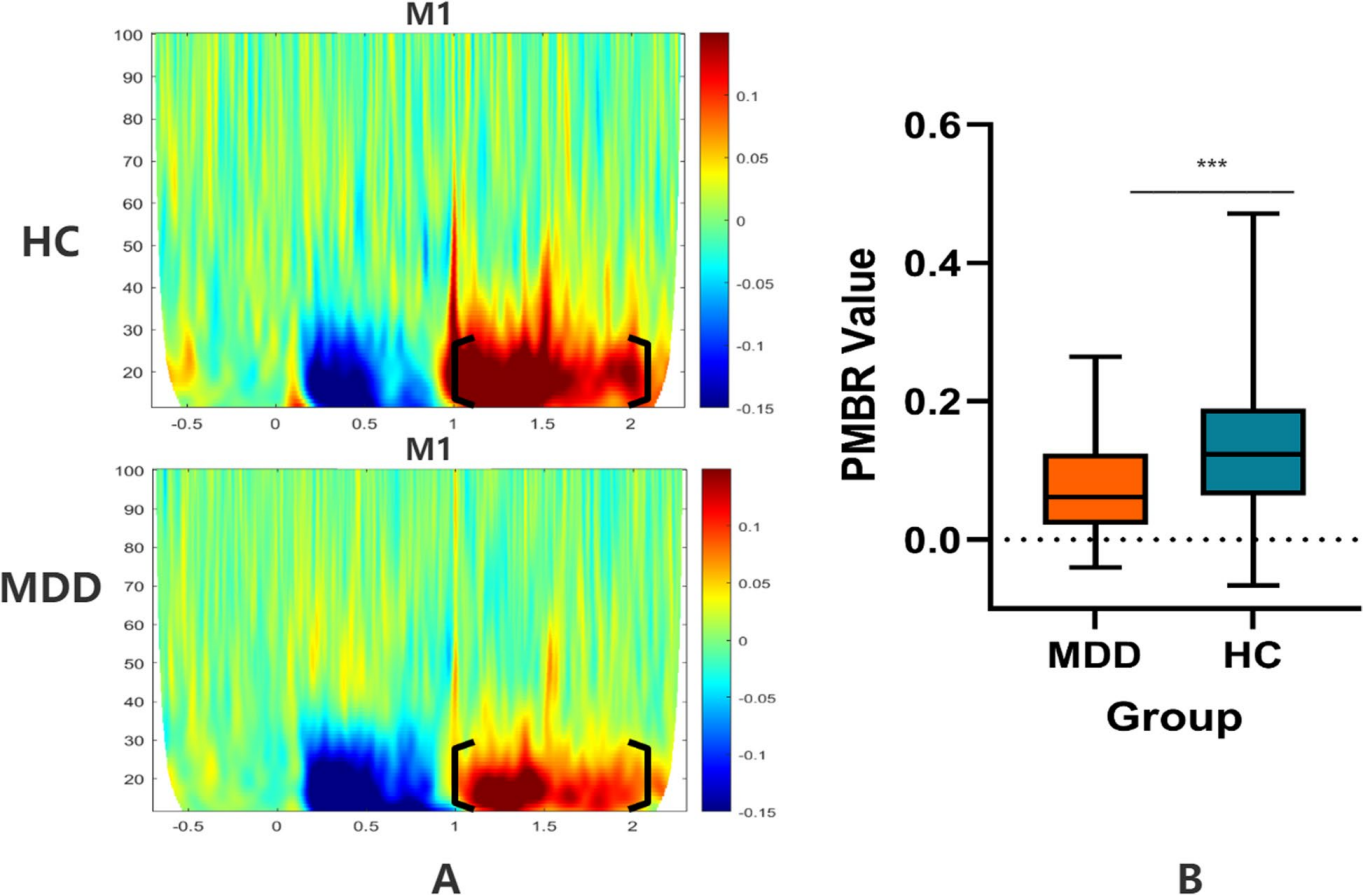
Differences of PMBR. **A** The time–frequency plots of the left M1 for the MDD and HC group. Both figures illustrate that oscillatory activity in the beta band (13–30 Hz) decreases before movement and subsequently rises after movement cessation. The MDD group had a similar but weaker response compare with HCs. According to the graphs, beta power reduces approximately 0.2 s after visual cues and increases around 1 s following visual stimuli. The colour scale depicts relative power changes in comparison to the baseline level (blue colour suggests a drop, while red colour suggests an increase). **B** The PMBR value in MDD and HC group. ***p < 0.001

Figure 4A shows the time–frequency representations of the left M1 for the MDD and HC groups. We observed that PMBR appeared following MRBD in the HC group, which has been well-established by the work of others. The MMD group experienced a similar but weaker response compared to the HC group. The graph shows that beta amplitude increases around 1 s following the visual stimulus. The PMBR value in the MDD group was lower than that in the HC group (*t* = 3.70, *p* < 0.001) (Table 2, Fig. 4B).

### Association between PMBR and clinical assessments

**Fig. 5:**
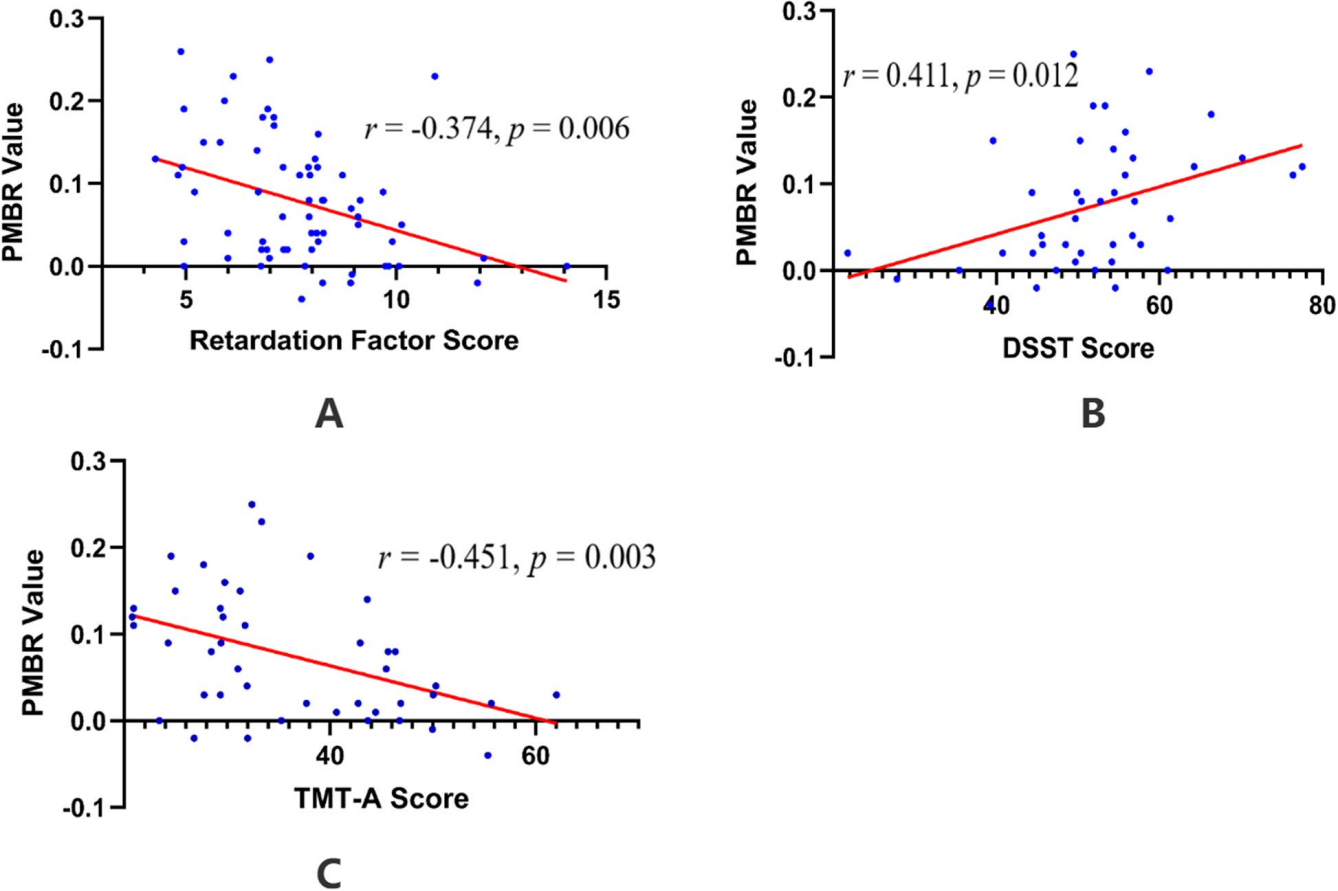
Correlations between PMBR and psychomotor alterations controlling for age, sex and education after FDR correction. **A** The PMBR of the left M1 negatively correlated with retardation factor scores. **B** The PMBR of the left M1 had a positive relationship with DSST scores. **C** The PMBR of the left M1 is negatively associated with TMT-A scores

In the MDD group, a two-tailed Pearson’s partial correlation was performed to compare the PMBR values with retardation factor scores. Psychomotor alterations are reflective of items relating to exhaustion, energy loss, or lack of attention in HAMD_17_. So, the retardation factor in HAMD_17_ can partially represent psychomotor alterations. In MDD patients, we particularly focused on the retardation factor scores, and we found that the retardation scores were inversely correlated with PMBR (*r* = -0.365, *p* = 0.002). Moreover, given that healthy ageing has an impact on beta oscillations [36–45], the correlations remained significant (*r* = -0.374, *p* = 0.006, FDR corrected) after controlling sex, age and education (Fig. 5A).

### Association between PMBR and neurocognitive assessments

In the MDD group, partial correlation analysis revealed a significant positive correlation between the DSST scores and PMBR values (*r* = 0.411, *p* = 0.012, FDR corrected). Moreover, the TMT-A scores were negatively associated with PMBR (*r* = -0.451, *p* = 0.003, FDR corrected). The VFT scores were positively correlated with PMBR (*r* = 0.348, *p* = 0.022). However, this effect is no longer significant after controlling sex, age and education, and performing FDR correction (*r* = 0.348, *p* = 0.06, FDR corrected). Figure 5B and C visualise the relationship between neurocognitive assessments and PMBR amplitude. There was a significant negative correlation between age and PMBR in HCs (*r* = -0.300, *p* = 0.015). However, age was not significantly correlated with PMBR in MDD patients.

## Discussion

Deficit in psychomotor function has been acknowledged as an essential feature of MDD for decades. However, despite its prevalence and impact, few studies have probed the neuronal mechanisms underlying these symptoms. In this study, we primarily explored the relevance of the abnormal motor-related oscillatory activity to the psychomotor alterations in patients with MDD using clinical assessments, neurocognitive tests and a simple visuomotor task during MEG recording. More specifically, MDD patients had slower psychomotor speed and attenuated PMBR activity than HCs. Furthermore, there was an association between psychomotor alterations and PMBR values in the MDD group. These findings support our earlier hypothesis that the strength of motor-related cortical oscillations impacts slowing mental and motor processes in MDD. In addition, there was a significant negative correlation between age and PMBR in HCs.

In line with earlier studies that MDD patients have diminished sensorimotor cortex activity [2], we found that MDD patients had lower PMBR in M1 at the end of movement than HCs in our visuomotor MEG study. Several explanations have been put forward to interpret PMBR. One of the theories is that PMBR is generated by the peripheral electrical stimulation of afferent pathways or somatosensory stimuli [66, 67], indicating afferent sensory feedback on the completion of the movement [30]. The somatosensory system is not only responsible for afferent input information but also actively communicates with the motor cortex during movement [68]. Poor sensory feedback may cause major problems with sensorimotor integration and reduce the accuracy of feedforward predictions[69]. The afferent sensory feedback could be applied to evaluate movement outcome, possibly impacting the following processing speed. The evidence from healthy individuals revealed that a “slowed movement state” displayed weaker sensorimotor beta dynamics [34] compared to fast movement [70]. More evidence has been provided that patients with motor abnormalities or processing speed slowing displayed attenuated PMBR. For example, Robson et al. found aberrant PMBR activity in SCH [50], as well as ASD [53–55] and PD [46–48]. Taken together with previous research, our results suggest that the reduced PMBR activity is characteristic of patients with aberrant psychomotor performance.

Furthermore, we did find that the PMBR activity is negatively associated with retardation factor scores in HAMD_17_, indicating that MDD patients with higher clinical retardation symptoms displayed weaker sensorimotor beta dynamics. The retardation factor in HAMD_17_ partly reflects psychomotor function [3], suggesting that retardation symptoms result from the dysfunction of motor-related brain areas in MDD [71, 72]. Northoff et al. aggregated available evidence in healthy subjects, MDD, BD and SCH, proposing three psychomotor mechanisms, including subcortical modulation of motor circuit, cortical modulation of motor circuit and balance between motor cortex activity and global cortical activity [2]. It is worth noting that all mechanisms lead to the motor cortex. In addition, a recent study found that reduced PMBR relates to the severity of mental disorganisation and impoverishment symptoms in SPD, which involves thought processing speed [52]. Collectively, these results suggested that the reduced PMBR activity in M1 is possibly one of the key nodes to illustrate the psychomotor alterations in MDD.

Moreover, our study also found that PMBR activity was associated with cognitive function in MDD. In our research, MDD patients performed worse on all three neurocognitive tests compared to HCs. Our findings are congruent with a prior meta-analysis, showing that MDD patients report impairments in motor speed, processing speed, executive functioning in tests with time limitations, and verbal fluency [73]. Consistently, the “time to peak” of the PMBR is associated with the DSST performance in multiple sclerosis (MS) patients [74]. Another study observed that PMBR was significantly correlated with DSST scores in patients with schizophrenia [51], providing strong evidence that PMBR activity could reflect information processing speed. Abnormal PMBR may represent aberrant sensory feedback. Patients with MDD thus have reduced feedback about the final outcomes of their motor actions, resulting in serious difficulties in sensorimotor integration and decreased certainty in the feedforward predictions that are based on the internal model. In addition, a reduced PMBR may reflect poor inhibition of motor cortex after movement termination in MDD. Overall, our study provides novel insights into neural mechanisms that are involved in psychomotor disturbances in MDD from the perspective of neural oscillations. This may lay the groundwork for novel biomarkers and individualised interventions for this difficult to treat feature of MDD.

### Limitations

Of note, there are some limitations in this study. First, scales to measure psychomotor alterations in MDD should be used in future studies, such as the Motor Agitation and Retardation Scale (MARS), the CORE index of melancholia, and the Salpetriere Retardation Rating Scale (SRRS) [75–77]. Second, PMBR is modulated by task duration [78] and is related to contraction force [79] and movement duration [80]. It will be better to control these factors in future studies. Thirdly, the current study only focused on M1. The impairments of cognitive function and clinical symptoms in MDD are caused not only by motor cortex dysfunction, but also by the coordination of distant brain areas. We will further explore the long-distance communication between brain regions in the next step.

## Conclusion

Our present MEG study has expanded on previous literature by demonstrating that motor-related beta oscillations are weaker in patients with MDD than HCs during tasks involving psychomotor processes, reflecting difficulties in sensorimotor integration. Moreover, the current results provide direct electrophysiological evidence that the reduced PMBR activity contributes to clinical symptoms and cognitive functions in MDD, identifying a potential electrophysiological biomarker for assessing PMR. These electrophysiological imaging results offer a “Spatiotemporal Psychopathology” insight into abnormal psychomotor function suffered by MDD patients.

## Data Availability

The datasets generated during the current study are not publicly available due to the subjects’ privacy, but are available from the corresponding author on reasonable request.

## Abbreviations

MDD: Major depressive disorder
PMR: Psychomotor retardation
BD: Bipolar disorder
SCH: Schizophrenia
CBF: Cerebral blood flow
M1: Primary motor cortex
MRBD: Movement-related beta reduction
PMBR: Post-movement beta rebound
PD: Parkinson's disease
SPD: Schizotypal personality disorder
ASD: Autism spectrum disorder
HAMD_17_: 17-Item Hamilton Rating Scale for Depression
TMT-A: Trail Making Test Part A
DSST: Digit Symbol Substitution Test
VFT: Verbal Fluency Test
MEG: Magnetoencephalography
HCs: Healthy controls
DSM-IV-TR: Diagnostic and Statistical Manual of Mental Disorders Fourth Edition, Text Revision
YMRS: Young Mania Rating Scale
LCMV: Linearly constrained minimum variance
AAL: Automated Anatomical Labeling
MS: Multiple sclerosis
MARS: Motor Agitation and Retardation Scale
SRRS: Salpetriere Retardation Rating Scale

## References

[1] Sobin C, Sackeim HA (1997). Psychomotor symptoms of depression. Am J Psychiatry.

[2] Northoff G, Hirjak D, Wolf RC, Magioncalda P, Martino M (2021). All roads lead to the motor cortex: psychomotor mechanisms and their biochemical modulation in psychiatric disorders. Mol Psychiatry.

[3] Bennabi D, Vandel P, Papaxanthis C, Pozzo T, Haffen E (2013). Psychomotor retardation in depression: a systematic review of diagnostic, pathophysiologic, and therapeutic implications. Biomed Res Int..

[4] Calugi S, Cassano GB, Litta A, Rucci P, Benvenuti A, Miniati M, Lattanzi L, Mantua V, Lombardi V, Fagiolini A (2011). Does psychomotor retardation define a clinically relevant phenotype of unipolar depression?. J Affect Disord.

[5] Gorlyn M, Keilp JG, Grunebaum MF, Taylor BP, Oquendo MA, Bruder GE, Stewart JW, Zalsman G, Mann JJ (2008). Neuropsychological characteristics as predictors of SSRI treatment response in depressed subjects. J Neural Transm (Vienna).

[6] Bruder GE, Alvarenga JE, Alschuler D, Abraham K, Keilp JG, Hellerstein DJ, Stewart JW, McGrath PJ (2014). Neurocognitive predictors of antidepressant clinical response. J Affect Disord.

[7] Buyukdura JS, McClintock SM, Croarkin PE (2011). Psychomotor retardation in depression: biological underpinnings, measurement, and treatment. Prog Neuropsychopharmacol Biol Psychiatry.

[8] Martino M, Magioncalda P, Conio B, Capobianco L, Russo D, Adavastro G, Tumati S, Tan Z, Lee HC, Lane TJ (2020). Abnormal Functional Relationship of Sensorimotor Network With Neurotransmitter-Related Nuclei via Subcortical-Cortical Loops in Manic and Depressive Phases of Bipolar Disorder. Schizophr Bull.

[9] Martino M, Magioncalda P, Huang Z, Conio B, Piaggio N, Duncan NW, Rocchi G, Escelsior A, Marozzi V, Wolff A (2016). Contrasting variability patterns in the default mode and sensorimotor networks balance in bipolar depression and mania. Proc Natl Acad Sci U S A.

[10] Mittal VA, Bernard JA, Northoff G (2017). What Can Different Motor Circuits Tell Us About Psychosis?. An RDoC Perspective Schizophr Bull.

[11] Videbech P, Ravnkilde B, Pedersen TH, Hartvig H, Egander A, Clemmensen K, Rasmussen NA, Andersen F, Gjedde A, Rosenberg R (2002). The Danish PET/depression project: clinical symptoms and cerebral blood flow A regions-of-interest analysis. Acta Psychiatr Scand.

[12] Narita H, Odawara T, Iseki E, Kosaka K, Hirayasu Y (2004). Psychomotor retardation correlates with frontal hypoperfusion and the Modified Stroop Test in patients under 60-years-old with major depression. Psychiatry Clin Neurosci.

[13] Mayberg HS, Lewis PJ, Regenold W, Wagner HN (1994). Paralimbic hypoperfusion in unipolar depression. J Nucl Med.

[14] Bracht T, Federspiel A, Schnell S, Horn H, Höfle O, Wiest R, Dierks T, Strik W, Müller TJ, Walther S (2012). Cortico-cortical white matter motor pathway microstructure is related to psychomotor retardation in major depressive disorder. PLoS One..

[15] Walther S, Hügli S, Höfle O, Federspiel A, Horn H, Bracht T, Wiest R, Strik W, Müller TJ (2012). Frontal white matter integrity is related to psychomotor retardation in major depression. Neurobiol Dis.

[16] Hickie I, Scott E, Mitchell P, Wilhelm K, Austin MP, Bennett B (1995). Subcortical hyperintensities on magnetic resonance imaging: clinical correlates and prognostic significance in patients with severe depression. Biol Psychiatry.

[17] Yin Y, Wang M, Wang Z, Xie C, Zhang H, Zhang H, Zhang Z, Yuan Y (2018). Decreased cerebral blood flow in the primary motor cortex in major depressive disorder with psychomotor retardation. Prog Neuropsychopharmacol Biol Psychiatry.

[18] Pfurtscheller G, Lopes da Silva FH (1999). Event-related EEG/MEG synchronization and desynchronization: basic principles. Clin Neurophysiol..

[19] Kilavik BE, Zaepffel M, Brovelli A, MacKay WA, Riehle A (2013). The ups and downs of β oscillations in sensorimotor cortex. Exp Neurol.

[20] Fingelkurts AA, Fingelkurts AA, Rytsälä H, Suominen K, Isometsä E, Kähkönen S (2006). Composition of brain oscillations in ongoing EEG during major depression disorder. Neurosci Res.

[21] Fingelkurts AA, Fingelkurts AA (2015). Altered structure of dynamic electroencephalogram oscillatory pattern in major depression. Biol Psychiatry.

[22] Jiang H, Popov T, Jylänki P, Bi K, Yao Z, Lu Q, Jensen O, van Gerven MA (2016). Predictability of depression severity based on posterior alpha oscillations. Clin Neurophysiol.

[23] Jiang H, Dai Z, Lu Q, Yao Z (2020). Magnetoencephalography resting-state spectral fingerprints distinguish bipolar depression and unipolar depression. Bipolar Disord.

[24] Jiang H, Hua L, Dai Z, Tian S, Yao Z, Lu Q, Popov T (2019). Spectral fingerprints of facial affect processing bias in major depression disorder. Soc Cogn Affect Neurosci.

[25] Wang H, Tian S, Yan R, Tang H, Shi J, Zhu R, Chen Y, Han Y, Chen Z, Zhou H (2022). Convergent and divergent cognitive impairment of unipolar and bipolar depression: A magnetoencephalography resting-state study. J Affect Disord.

[26] Parkkonen E, Laaksonen K, Piitulainen H, Parkkonen L, Forss N (2015). Modulation of the ∽20-Hz motor-cortex rhythm to passive movement and tactile stimulation. Brain Behav..

[27] Jurkiewicz MT, Gaetz WC, Bostan AC, Cheyne D (2006). Post-movement beta rebound is generated in motor cortex: evidence from neuromagnetic recordings. Neuroimage.

[28] Miller KJ, Leuthardt EC, Schalk G, Rao RP, Anderson NR, Moran DW, Miller JW, Ojemann JG (2007). Spectral changes in cortical surface potentials during motor movement. J Neurosci.

[29] Solis-Escalante T, Müller-Putz GR, Pfurtscheller G, Neuper C (2012). Cue-induced beta rebound during withholding of overt and covert foot movement. Clin Neurophysiol.

[30] Houdayer E, Labyt E, Cassim F, Bourriez JL, Derambure P (2006). Relationship between event-related beta synchronization and afferent inputs: analysis of finger movement and peripheral nerve stimulations. Clin Neurophysiol.

[31] Tan H, Wade C, Brown P (2016). Post-Movement Beta Activity in Sensorimotor Cortex Indexes Confidence in the Estimations from Internal Models. J Neurosci.

[32] Hervault M, Zanone PG, Buisson JC, Huys R (2021). Cortical sensorimotor activity in the execution and suppression of discrete and rhythmic movements. Sci Rep.

[33] Cao L, Hu Y (2016). Beta Rebound in Visuomotor Adaptation: Still the Status Quo?. J Neurosci.

[34] Leriche RB, Jackson N, Peterson K, Aspandiar Z, Hufnagel V, Swann NC. Reduced sensorimotor beta dynamics could represent a "slowed movement state" in healthy individuals. Neuropsychologia. 2022:108276. 10.1016/j.neuropsychologia.2022.108276. 10.1016/j.neuropsychologia.2022.108276 35636633

[35] Heinrichs-Graham E, Wilson TW, Santamaria PM, Heithoff SK, Torres-Russotto D, Hutter-Saunders JA, Estes KA, Meza JL, Mosley RL, Gendelman HE (2014). Neuromagnetic evidence of abnormal movement-related beta desynchronization in Parkinson's disease. Cereb Cortex.

[36] Gaetz W, Macdonald M, Cheyne D, Snead OC (2010). Neuromagnetic imaging of movement-related cortical oscillations in children and adults: age predicts post-movement beta rebound. Neuroimage.

[37] Bardouille T, Bailey L (2019). Evidence for age-related changes in sensorimotor neuromagnetic responses during cued button pressing in a large open-access dataset. Neuroimage.

[38] Heinrichs-Graham E, Wilson TW (2016). Is an absolute level of cortical beta suppression required for proper movement? Magnetoencephalographic evidence from healthy aging. Neuroimage.

[39] Xifra-Porxas A, Niso G, Larivière S, Kassinopoulos M, Baillet S, Mitsis GD, Boudrias MH (2019). Older adults exhibit a more pronounced modulation of beta oscillations when performing sustained and dynamic handgrips. Neuroimage..

[40] Rossiter HE, Davis EM, Clark EV, Boudrias MH, Ward NS (2014). Beta oscillations reflect changes in motor cortex inhibition in healthy ageing. Neuroimage.

[41] Walker S, Monto S, Piirainen JM, Avela J, Tarkka IM, Parviainen TM, Piitulainen H (2020). Older Age Increases the Amplitude of Muscle Stretch-Induced Cortical Beta-Band Suppression But Does not Affect Rebound Strength. Front Aging Neurosci.

[42] Gehringer JE, Arpin DJ, VerMaas JR, Trevarrow MP, Wilson TW, Kurz MJ (2019). The Strength of the Movement-related Somatosensory Cortical Oscillations Differ between Adolescents and Adults. Sci Rep.

[43] Groth CL, Singh A, Zhang Q, Berman BD, Narayanan NS (2021). GABAergic Modulation in Movement Related Oscillatory Activity: A Review of the Effect Pharmacologically and with Aging. Tremor Other Hyperkinet Mov (N Y).

[44] Gehringer JE, Arpin DJ, Heinrichs-Graham E, Wilson TW, Kurz MJ (2019). Practice modulates motor-related beta oscillations differently in adolescents and adults. J Physiol.

[45] Trevarrow MP, Kurz MJ, McDermott TJ, Wiesman AI, Mills MS, Wang YP, Calhoun VD, Stephen JM, Wilson TW (2019). The developmental trajectory of sensorimotor cortical oscillations. Neuroimage.

[46] Hall SD, Prokic EJ, McAllister CJ, Ronnqvist KC, Williams AC, Yamawaki N, Witton C, Woodhall GL, Stanford IM (2014). GABA-mediated changes in inter-hemispheric beta frequency activity in early-stage Parkinson's disease. Neuroscience.

[47] Boon LI, Geraedts VJ, Hillebrand A, Tannemaat MR, Contarino MF, Stam CJ, Berendse HW (2019). A systematic review of MEG-based studies in Parkinson's disease: The motor system and beyond. Hum Brain Mapp.

[48] Heinrichs-Graham E, Santamaria PM, Gendelman HE, Wilson TW (2017). The cortical signature of symptom laterality in Parkinson's disease. Neuroimage Clin.

[49] Gascoyne LE, Brookes MJ, Rathnaiah M, Katshu M, Koelewijn L, Williams G, Kumar J, Walters JTR, Seedat ZA, Palaniyappan L (2021). Motor-related oscillatory activity in schizophrenia according to phase of illness and clinical symptom severity. Neuroimage Clin..

[50] Robson SE, Brookes MJ, Hall EL, Palaniyappan L, Kumar J, Skelton M, Christodoulou NG, Qureshi A, Jan F, Katshu MZ (2016). Abnormal visuomotor processing in schizophrenia. Neuroimage Clin.

[51] Rathnaiah M, Liddle EB, Gascoyne L, Kumar J, Faruqi C, Kelly C, Gill M, Robson S, Brookes M, Zia UlHaqKatshu M (2020). Quantifying the Core Deficit in Classical Schizophrenia. Schizophr Bull Open..

[52] Hunt B, Liddle E, Gascoyne L, Magazzini L, Routley B, Singh K, Morris P, Brookes M, Liddle P (2019). Attenuated Post-Movement Beta Rebound Associated With Schizotypal Features in Healthy People. Schizophr Bull.

[53] An KM, Ikeda T, Hasegawa C, Yoshimura Y, Tanaka S, Saito DN, Yaoi K, Iwasaki S, Hirosawa T, Jensen O (2021). Aberrant brain oscillatory coupling from the primary motor cortex in children with autism spectrum disorders. Neuroimage Clin..

[54] Gaetz W, Rhodes E, Bloy L, Blaskey L, Jackel C, Brodkin E, Waldman A, Embick D, Hall S, Roberts T (2020). Evaluating motor cortical oscillations and age-related change in autism spectrum disorder. Neuroimage..

[55] Honaga E, Ishii R, Kurimoto R, Canuet L, Ikezawa K, Takahashi H, Nakahachi T, Iwase M, Mizuta I, Yoshimine T (2010). Post-movement beta rebound abnormality as indicator of mirror neuron system dysfunction in autistic spectrum disorder: an MEG study. Neurosci Lett.

[56] Young RC, Biggs JT, Ziegler VE, Meyer DA (1978). A rating scale for mania: reliability, validity and sensitivity. Br J Psychiatry.

[57] Mowbray RM (1972). The Hamilton Rating Scale for depression: a factor analysis. Psychol Med.

[58] Hamilton M (1967). Development of a rating scale for primary depressive illness. Br J Soc Clin Psychol.

[59] Bowie CR, Harvey PD (2006). Administration and interpretation of the Trail Making Test. Nat Protoc.

[60] Baune BT, Brignone M, Larsen KG (2018). A Network Meta-Analysis Comparing Effects of Various Antidepressant Classes on the Digit Symbol Substitution Test (DSST) as a Measure of Cognitive Dysfunction in Patients with Major Depressive Disorder. Int J Neuropsychopharmacol.

[61] van Hoof JJ, Hulstijn W, van Mier H, Pagen M (1993). Figure drawing and psychomotor retardation: preliminary report. J Affect Disord.

[62] Han YL, Dai ZP, Ridwan MC, Lin PH, Zhou HL, Wang HF, Yao ZJ, Lu Q (2020). Connectivity of the Frontal Cortical Oscillatory Dynamics Underlying Inhibitory Control During a Go/No-Go Task as a Predictive Biomarker in Major Depression. Front Psychiatry.

[63] Oostenveld R, Fries P, Maris E, Schoffelen JM (2011). FieldTrip: Open source software for advanced analysis of MEG, EEG, and invasive electrophysiological data. Comput Intell Neurosci..

[64] Van Veen BD, van Drongelen W, Yuchtman M, Suzuki A (1997). Localization of brain electrical activity via linearly constrained minimum variance spatial filtering. IEEE Trans Biomed Eng.

[65] Tzourio-Mazoyer N, Landeau B, Papathanassiou D, Crivello F, Etard O, Delcroix N, Mazoyer B, Joliot M (2002). Automated anatomical labeling of activations in SPM using a macroscopic anatomical parcellation of the MNI MRI single-subject brain. Neuroimage.

[66] Salenius S, Schnitzler A, Salmelin R, Jousmäki V, Hari R (1997). Modulation of human cortical rolandic rhythms during natural sensorimotor tasks. Neuroimage.

[67] Gaetz W, Cheyne D (2006). Localization of sensorimotor cortical rhythms induced by tactile stimulation using spatially filtered MEG. Neuroimage.

[68] Umeda T, Isa T, Nishimura Y (2019). The somatosensory cortex receives information about motor output. Sci Adv..

[69] Trevarrow MP, Reelfs A, Baker SE, Hoffman RM, Wilson TW, Kurz MJ (2022). Spinal cord microstructural changes are connected with the aberrant sensorimotor cortical oscillatory activity in adults with cerebral palsy. Sci Rep.

[70] Zhang X, Li H, Xie T, Liu Y, Chen J, Long J (2020). Movement speed effects on beta-band oscillations in sensorimotor cortex during voluntary activity. J Neurophysiol.

[71] Martinot M, Bragulat V, Artiges E, Dollé F, Hinnen F, Jouvent R, Martinot J (2001). Decreased presynaptic dopamine function in the left caudate of depressed patients with affective flattening and psychomotor retardation. Am J Psychiatry.

[72] Naismith S, Hickie I, Ward PB, Turner K, Scott E, Little C, Mitchell P, Wilhelm K, Parker G (2002). Caudate nucleus volumes and genetic determinants of homocysteine metabolism in the prediction of psychomotor speed in older persons with depression. Am J Psychiatry.

[73] Semkovska M, Quinlivan L, O'Grady T, Johnson R, Collins A, O'Connor J, Knittle H, Ahern E, Gload T (2019). Cognitive function following a major depressive episode: a systematic review and meta-analysis. Lancet Psychiatry.

[74] Barratt EL, Tewarie PK, Clarke MA, Hall EL, Gowland PA, Morris PG, Francis ST, Evangelou N, Brookes MJ (2017). Abnormal task driven neural oscillations in multiple sclerosis: A visuomotor MEG study. Hum Brain Mapp.

[75] Jouvent R, Frechette D, Binoux F, Lancrenon S, des Lauriers A (1980). Retardation in depressive states : elaboration of a quantitative rating scale (author's transl). Encephale..

[76] Sobin C, Mayer L, Endicott J (1998). The motor agitation and retardation scale: a scale for the assessment of motor abnormalities in depressed patients. J Neuropsychiatry Clin Neurosci.

[77] Parker G, Hadzi-Pavlovic D, Austin MP, Mitchell P, Wilhelm K, Hickie I, Boyce P, Eyers K (1995). Sub-typing depression, I. Is psychomotor disturbance necessary and sufficient to the definition of melancholia?. Psychol Med..

[78] Pakenham DO, Quinn AJ, Fry A, Francis ST, Woolrich MW, Brookes MJ, Mullinger KJ (2020). Post-stimulus beta responses are modulated by task duration. Neuroimage..

[79] Fry A, Mullinger KJ, O'Neill GC, Barratt EL, Morris PG, Bauer M, Folland JP, Brookes MJ (2016). Modulation of post-movement beta rebound by contraction force and rate of force development. Hum Brain Mapp.

[80] Feingold J, Gibson DJ, DePasquale B, Graybiel AM (2015). Bursts of beta oscillation differentiate postperformance activity in the striatum and motor cortex of monkeys performing movement tasks. Proc Natl Acad Sci U S A.

